# Nurses’ Assessments Versus Patients’ Self-Assessments of Postoperative Pain: Knowledge and Skills of Nurses for Effective Pain Management

**DOI:** 10.3390/ijerph20095678

**Published:** 2023-04-28

**Authors:** Marija Kadović, Stipe Ćorluka, Stjepan Dokuzović

**Affiliations:** 1 Faculty of Medicine, Josip Juraj Strossmayer University of Osijek, 31000 Osijek, Croatia; 2 Spinal Surgery Division, Department of Traumatology, University Hospital Centre Sestre Milosrdnice, 10000 Zagreb, Croatia; stipe.corluka@kbcsm.hr (S.Ć.); stjepan.dokuzovic@kbcsm.hr (S.D.)

**Keywords:** acute postoperative pain, perioperative nursing, nursing assessment, pain assessment, pain intensity, pain management, pain scale, quality of healthcare, health care professionals

## Abstract

Postoperative pain is the most common form of acute pain. Nurses contribute to effective pain management with their knowledge and skills. The aims of this research were to examine differences between nurses’ assessments and patients’ self-assessments of postoperative pain, differences in the mentioned (self) assessments with respect to characteristics of both groups of respondents, and the correlation between the NRS and the VRS scale. The study included 103 nurses employed at a hospital and 103 patients treated in the surgical departments after the surgical procedures. Data were collected using the standardized Numerical rating scale (NRS) and Verbal rating score (VRS). The median of patients’ self-assessments of pain intensity on the NRS scale was 4, while the nursing assessment of patients’ pain was 3, with no significant difference (*p* = 0.083). No significant differences were found on the VRS scale between nurse assessments and patient self-assessments of current pain intensity. The pain was described as moderate by 35% of participants, including 35.9% nurses and 35% patients. Significant positive correlations were recorded between values on the VRS and NRS scales for nurses (Rho = 0.812; *p* < 0.001) and patients (Rho = 0.830; *p* < 0.001). The results of this study may have implications for the improvement of postoperative pain management protocols, with regular use of pain assessment scales and individualization of analgesic prescriptions.

## 1. Introduction

### 1.1. Postoperative Pain

Postoperative pain has long been ignored or inadequately treated [1]. Pain assessment is the first and most important step towards adequate pain relief and addressing the problem of pain. After reporting pain, patients in hospital care often do not have the ability to objectively describe their pain and receive the therapy they truly need [1]. Several studies have shown differences between a patient’s self-assessment of pain and the assessment made by nurses, which can lead to incorrect information being conveyed to the physician regarding the level of the patient’s pain and result in the administration of inadequate amounts of analgesics [2,3]. Postoperative pain is the most common form of acute pain, characterised by constant pain associated with the surgical procedure, typically near the site of the operation [1]. Acute exacerbation of pain in addition to underlying pain occurs with coughing, getting out of bed, physical therapy, and dressing changes and is a self-limiting condition that typically progressively improves over a relatively short period of time [4]. Intense nociception and neural hypersensitivity after surgical incision are caused and sustained by afferent impulses from sensitised nociceptors during the postoperative period, not just due to the initial injury [5]. Therefore, in the treatment of postoperative pain, the timing, effectiveness, and duration of analgesic procedures are crucial. In addition to pain relief, proper postoperative pain treatment plays an important role in improving patient recovery and reducing hospital stays [6]. Meissner and colleagues, speaking about the effectiveness and safety of postoperative pain management in clinical hospitals, state that 80% of patients experience moderate to severe postoperative pain [7]. The International Association for the Study of Pain (IASP) states that clinicians are not sufficiently educated on the need for acute pain treatment and the consequences of untreated pain [8]. In addition, a high percentage of European hospitals do not yet have written algorithms and protocols to manage postoperative pain, do not assess the intensity of pain, and treat pain only at the patient’s request [9]. Untreated acute postoperative pain is the strongest trigger for the stress response that initiates vitally threatening cascades of metabolic and inflammatory responses [10]. The study by Kehlet shows that increased and prolonged sympathetic, neurohumoral, and immune responses to acute pain inadequately treated in daily clinical practise lead to delayed surgical wound healing, increased frequency of pulmonary complications, thromboembolic incidents, and increased frequency of cardiovascular complications, especially coronary incidents [11]. Education of all healthcare staff, especially nurses who assess postoperative pain, and taking individual responsibility for patient care are currently the only ways to improve the assessment and treatment of postoperative pain.

### 1.2. Assessment and Self-Assessment of Pain

Assessment of pain intensity level is a key component in providing effective pain treatment. The systematic process of evaluation, measurement, and re-assessment improves the ability of nurses and healthcare teams, in general, to reduce the experience of pain in patients; increase comfort and satisfaction during hospital stays; and improve physiological, psychological, and physical functions after surgery [11]. To avoid differences in pain assessment, nurses need to use concrete data collection procedures (objectification of pain). In addition to the quantitative assessment, the nurses also carry out a qualitative assessment of the pain, which includes a description of the pain, duration, frequency, time of occurrence, and reaction to the pain [11]. Assessing pain in patients without predetermined measuring instruments to assess pain characteristics can be subjective and challenging, but there are some signs and symptoms that may indicate that the patient is experiencing pain, such as the patient’s facial expression [4]. Influential factors, in addition to external and physical factors, can also be gender-related [12]. There are certain differences in pain experience between men and women. These differences are attributed to biological differences between sexes and the influence of psychosocial factors [12]. The quality of a nurse’s assessment of a patient’s pain can be influenced by the nurse’s individual education, personal experience with pain, the type of surgical procedure the patient underwent, the patient’s age, the number of days since the procedure, the patient’s habits, or the patient’s cultural background [13,14]. Such an assessment can also help monitor the patient’s progress and evaluate the effectiveness of pain therapies.

There are several types of instruments [1] to assess postoperative pain; patient numerical and verbal pain scales are used most frequently in surgical hospital departments and are relevant to the development of pain treatment guidelines [4]. Pain can be evaluated at physiological, subjective, and behavioural levels [15]. Subjective assessment involves the patient’s statements about the location, quality, and intensity of pain, which are sometimes inconsistent with other indicators of painful experience and disproportionate to the severity of the disease [16]. Giusti et al. note in their study that patient self-reported pain assessment is considered the gold standard of assessment and is the most accurate measurement tool [15]. Self-assessment of pain can result in inaccuracy if the patient is influenced by changes in mood, sleep disturbances, or medications [17]. Nurses, on the other hand, may be sceptical of the patient’s self-assessment of pain, as they create their own standards of what is acceptable and when and how patients should express their pain [17].

Subjective pain assessment includes one-dimensional and multidimensional tools. The most commonly used one-dimensional tools in the assessment of acute pain intensity are visual analogue scales; verbal, graphic, and numeric rating scales; pain flow diagrams; computer graphics; and pictorial scales [18,19], as such scales are fast, accurate, easy to use, and understandable. In the acute pain model, it is recommended to systematically introduce a numeric or visual scale when assessing the intensity of postoperative acute pain [20]. In Dijkers’ longitudinal study [21], significant differences in the use of VRS and NRS scales were observed among neurosurgical patients, as well as a lack of understanding of the meaning of the VRS scale among certain patients. Multidimensional tools [19] can provide information on the qualitative and quantitative aspects of pain and may be useful in cases of suspected neuropathic pain.

### 1.3. Individual Approach to Pain Management

Treatment of acute pain requires close interdisciplinary collaboration in the planning and implementation of treatment [22,23]. Effective postoperative pain management also requires quality communication with the patient in preparation for surgery and access to the patient and their family in the postoperative period [24]. Despite the preoperative conversation, it is still standard practice in surgical departments for the doctor to prescribe a fixed dose of analgesics “as needed”, regardless of measurable records of pain intensity in medical documentation [25]. In this context, the importance of medical documentation is considered when there is a lack of coordination and the possibility of errors in communication among healthcare professionals working in shifts [4]. The individualised approach to pain management faces obstacles in systems where there is a lack of healthcare personnel, overcrowding, and high turnover of patients in departments. In such cases, employees can be overwhelmed with work, which can reduce the amount of time they can dedicate to assessing patient pain properly.

Pain, as one of the most common patient symptoms encountered by nurses in their practice, has been analysed and described in theories by well-known nurses. The theory of unpleasant symptoms is the first midrange theory that deals with the assessment of multiple symptoms and, as such, provides a broader view of the interrelationship between symptoms and their outcomes [26]; this theory was used as the basis of this study. Scientific literature review shows that despite numerous studies on postoperative pain management [12,22,24], there is still an insufficient number of new studies examining the level of pain assessment by nurses and patient self-assessment of pain. This deficit can affect the global understanding of the importance of individualising postoperative pain treatment, where the objectification of assessed postoperative pain is necessary to prevent subanalgesia and inadequate pain management in general. Therefore, the aims of this research are to examine the following: (a) differences between nurses’ assessments and patients’ self-assessments of postoperative pain, (b) differences in the mentioned (self) assessments with respect to characteristics of both groups of respondents (gender, age, nursing level of education, and work experience), and (c) the correlation between the NRS and the VRS scale.

## 2. Materials and Methods

### 2.1. Study Design

In this study, a quantitative research approach was applied with the use of two validated scales and measurement methods. Data were collected from respondents only once, in two different time periods, which indicates a cross-sectional design. Given that the goals of this research were primarily focused on examining differences between nursing assessments and patient self-assessments of postoperative pain and examining correlations between the two applied scales, this study can be described as a quantitative, cross-sectional correlational study.

### 2.2. Setting

This study was conducted at the Clinical Hospital Center in the Republic of Croatia in four departments where surgical patients are treated (Abdominal surgery, Urology, Orthopedics, and Otorhinolaryngology). The study was carried out in two phases, including a preoperative examination of the patient on sociodemographic data as well as expectations and fears about pain after surgery, alongside a simultaneous assessment of pain by nurses and self-assessment of postoperative pain by patients in the mentioned health institution. The selection criteria for the healthcare institution included the following: (a) the healthcare institution in our study is the central and largest healthcare institution in this region of Croatia, (b) the healthcare institution belongs to the first category according to the Croatian classification (a national hospital with at least three clinics where the most complex diagnostic and therapeutic procedures are performed), (c) approval of the Hospital Board of Directors to conduct the study, (d) the institution is a teaching base of the faculty within which the researchers conduct the study. The criterion for selecting the hospital departments for the study meant that 24-h service was provided.

### 2.3. Respondents

The study sample consisted of working nurses and patients who were hospitalised at the Clinical Hospital Centre. Inclusion criteria for both samples were as follows: the respondents are (a) over 18 years of age, (b) read and understand the Croatian language, and (c) voluntarily participate in the study. Of the total of 223 respondents from both samples, 206 completed the questionnaires (response rate of 91%). From the nursing staff group, the response rate was 100%, while from the patient group it was 85.8%, because 17 patients did not meet the inclusion criteria on the first postoperative day. To detect a medium effect (d = 0.5) in the difference of numerical variables between nurses and patients with a significance level of 0.05 and a power of 0.80, the minimum required sample size was 128 respondents (64 per group) (G*Power 3.1.9.4).

Additional inclusion criteria for the sample of nurses were as follows: (a) respondents are nurses permanently employed at the mentioned health institution, (b) respondents provide direct healthcare to hospitalised patients; exclusion criteria included nurses employed in the outpatient clinic for day surgery and in the operating room. Additional inclusion criteria for the sample of patients were the following: (a) hospitalised patients in surgical departments after simple elective surgeries (Tonsillectomy, Cholecystectomy, Hemorrhoidectomy, Inguinal hernia repair, low back pain surgery, Arthroscopy, Prostatectomy,) on the first postoperative day; (b) fully conscious and communicative, without obstacles in pronunciation (tube, cannula or muteness); (c) respondents have a predefined dose of analgesics on the Therapy List, which they can receive “on demand”. The exclusion criteria were as follows: patients operated on in a day surgery clinic (discharged on the first day after surgery); respondents on continuous permanent analgesia, with whom verbal communication cannot be established or with obstacles in pronunciation.

### 2.4. Instruments

The questionnaire used in this study included two validated standardised scales: (a) Numerical rating scale (NRS) [27] and (b) Verbal rating score (VRS) [28]. Both scales are publicly available and free for use by all nurses in all healthcare institutions in the Republic of Croatia. Therefore, both scales have been translated and are in clinical use as part of the national uniform nursing documentation, namely, the unique nursing documentation was created by the Croatian Council of Nurses and approved by the Ministry of Health of the Republic of Croatia [29]. The introductory part of the questionnaire was different for the group of nursing staff and contained questions about sociodemographic characteristics (sex, age, level of education, total duration of employment, working hours in shifts). The introductory part of the patient questionnaire contained data on age and gender. The structured standardised NRS scale, written in Croatian [29], allows patients to rate their pain from 1 to 10. The number 0 indicates a pain-free state, from 1 to 3 is moderate pain, from 4 to 6 is moderately tolerable pain, and from 7 to 10 is very severe to unbearable pain. The scale is applicable when working with children and the elderly. The disadvantage is that it is difficult to use for people with hearing or cognitive impairments [30]. The second standardised scale used in this study is VRS, which is a one-dimensional tool for measuring pain that is suitable for all conscious patients with stable cognitive patterns. It evaluates pain in five categories when patients mark one of the simply offered descriptions of pain intensity (“none”; “mild”; “moderate”; “severe”; or “worst pain”). Its disadvantage is that it avoids the description of pain and has a limited number of categories and descriptions [31].

### 2.5. Data Collection

Data were collected over two months through two phases. In the first phase, during admission of the patient to the surgical department, patients were asked if they wanted to participate in this anonymous study and signed an informed consent form to participate. The patients then completed the first part of the questionnaire, which included sociodemographic data. Furthermore, the researchers instructed the patients to inform the nurse of any pain on the first postoperative day using only the sentence “nurse, I am in pain”, along with the usual physical expressions of pain (moaning, groaning, grimacing). This formulation was used exclusively for research purposes to assess nursing pain objectification skills with as little verbal input as possible from the patient.

The surveyed nurses were also asked to anonymously participate in the study, for which they signed informed consent. In the first phase of data collection, they completed a section of the questionnaire that included sociodemographic questions. The researchers instructed them on the statement the patient would make when pain first appeared on the first postoperative day. After the surgical procedure, if the patient met the inclusion criteria, they could proceed to the second phase of the questionnaire completion. On the first postoperative day, when the patient informed the nurse for the first time that they were in pain, she assessed their pain level using the NRS and VRS scales based on the patient’s appearance and behaviour. Afterward, the patient self-assessed the intensity of their pain on the NRS and VRS scales. After completing the second phase of data collection, the patient received prescribed analgesia according to the standard pain management procedure in the hospital.

### 2.6. Ethical Consideration

Participation in the study was voluntary and anonymous. Along with the questionnaire, the respondents also received an introductory text containing information about the details of the study (objective, procedure, confidentiality, rights, and voluntariness). Respondents confirmed their voluntary participation in the study by signing a questionnaire and completing it for the researcher. The respondents had the right to withdraw from the study without consequences. The anonymity of the respondents was guaranteed, i.e., it was not possible to determine their identity from their answers. Only researchers had access to the study data. All procedures of translation of applied NRS and VRS scales are under the jurisdiction of the Croatian Council of Nurses and approved by the Ministry of Health of the Republic of Croatia [29]. The study was conducted in accordance with the approval of the Ethics Committee (No: 01–1721).

### 2.7. Data Analysis

The numerical data were described using basic measures of central tendency and dispersion. The normality of the distribution of the observed numerical variables was tested using the Kolmogorov–Smirnov test. Categorical variables were described using absolute and relative frequencies. The Mann–Whitney test was used to compare two independent groups when the data were not normally distributed. The Kruskal–Wallis test was used for more than two independent groups. Differences among categorical variables were tested using Fisher’s exact test. The correlation between numerical variables was measured using Spearman’s correlation coefficient (Rho). A significance level of α = 0.05 was chosen to evaluate the significance of the results. The computer software used for statistical analysis was MedCalc^®^ Statistical Software version 20.100 (MedCalc Software Ltd., Ostend, Belgium) and SPSS Statistics for Windows, Version 23.0 (Released 2015. IBM. Armonk, NY, USA: IBM Corp.).

## 3. Results

### 3.1. Sociodemographic Characteristics of Respondents

A total of 206 participants took part in this study, including 103 (50%) nurses and 103 (50%) patients (Table 1). The sample of nurses consisted of 95 (92%) women and 8 (8%) men with a median age of 39 years (interquartile range = 30–46). The median length of work experience for nurses was 20 years (interquartile range = 7–29) (Table 1). Among the patients, there were 91 (44%) men and 115 (56%) women, with a median age of 49 years (interquartile range = 36–67).

### 3.2. Nurse Assessment and Patient Self-Assessment of Postoperative Pain on the Numerical Rating Scale (NRS)

**Figure 1:**
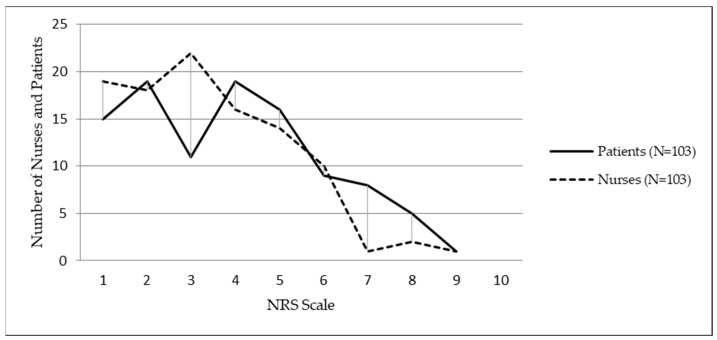
Nurses’ assessments and patient self-assessments of postoperative pain on the NRS scale.

The overall mean value (median) of patients’ self-assessments of pain intensity on the NRS scale was 4 (interquartile range 2–5), while the nursing assessment of patient pain was 3 (interquartile range 2–5), with no significant difference (Mann–Whitney test, *p* = 0.083). The distribution of the nurse and patient respondents with respect to the assigned numerical values of the assessed and self-assessed pain on the NRS scale is shown in Figure 1.

There was no significant difference in the mean values of nurses’ assessments and patient self-assessments of pain on the NRS scale with respect to any characteristic of both groups of participants (Table 2).

### 3.3. Nurses’ Assessments and Patient Self-Assessments of Current Pain Intensity on the Verbal Rating Scale (VRS)

**Figure 2:**
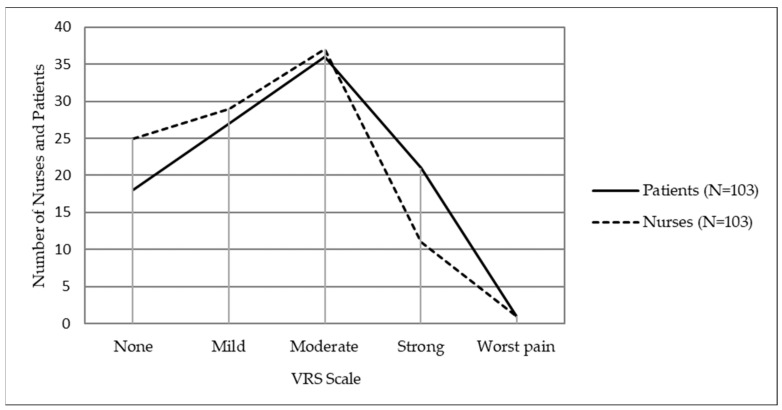
Nurse assessments and patient self-assessments of postoperative pain intensity on the NRS scale.

The distribution of nurse and patient participants with respect to assessed and self-assessed descriptions of the patient’s current pain intensity on the VRS scale is shown in Figure 2.

No significant differences were found on the VRS scale between nurse assessments and patient self-assessments of current pain intensity for any description (Table 3). Pain was described as moderate by 73 (35%) participants, including 37 (35.9%) nurses and 36 (35%) patients.

In the group of patients, mild pain was self-assessed by 11 (24.4%) men and 16 (27.6%) women, while strong pain was described by 8 (17.8%) men and 13 (22.4%) women, without a significant difference (*p* = 0.68) according to gender (Table 4).

In the group of nurses, no statistically significant differences were found in the pain descriptions assessed on the VRS scale with respect to sex, level of education, or duration of work experience (Table 4). The majority of nurses, specifically 37 (36%), assessed the patient’s pain as “unpleasant pain”.

Significant positive correlations were recorded between values on the VRS and NRS scales for both nurses (Rho = 0.812; *p* < 0.001) and patients (Rho = 0.830; *p* < 0.001).

## 4. Discussion

The purpose of this research was to gain insight into the differences between nursing assessments and patients’ self-assessments of postoperative pain in the mentioned health institution. Additionally, this study aimed to examine the differences in the mentioned (self) assessments with respect to characteristics of both groups of respondents and to examine the correlation between the NRS and VRS scales.

### 4.1. Differences between Nurses’ Assessments and Patient Self-Assessments of Postoperative Pain

The results of this study indicate that there is no significant difference in the overall mean value of pain assessed by nurses on the NRS scale, which is 3 (interquartile range 2–5), and the patient’s self-assessment of pain, which is 4 (interquartile range 2–5). Furthermore, there was no significant difference between nurses’ assessments and patients’ self-assessments of the current intensity of postoperative pain on the VRS scale.

Similar results were reported by a research team from Norway. They conducted a scoping review of multiple published studies and showed that achieving consistent pain assessment between patients and nurses on numerical and verbal pain scales can ensure better treatment of postoperative pain due to similar levels of understanding of pain by nurses and patients [32]. They also found that the relationship between the patient and the nurse is an important factor in how hospitalised patients assess and report their postoperative pain, and that the patient’s sense of inconsistency in how nurses conduct pain evaluation can lead to inadequate self-assessment of pain [32]. A more accurate pain assessment can result in a better selection of pain relief medication and the determination of optimal doses [33]. In the study by Kehlet and colleagues, it was noted that precise pain assessment by nurses ensures continuous pain management for patients in line with their needs [11]. This ensures effective pain control and reduces the risk of complications caused by uncontrolled pain, and patients feel more satisfied when they receive timely and adequate pain therapy [10,34]. Comparison of the pain intensity assessment between nurses and patients saves time for nurses and other healthcare workers by allowing them to quickly identify the level of pain the patient is experiencing and individualise the patient’s needs for acute pain medication. This improves the quality of the entire healthcare system [1]. Proven beneficial effects of acute postoperative pain treatment include the following [11]: earlier discharge of patients from intensive care units, shorter total duration of treatment, fewer serious complications that significantly prolong treatment time, more efficient use of healthcare staff’s working hours, more rational use of expensive hospital equipment, fewer days of physical incapacity for work, higher patient satisfaction, and reduced frequency of chronic pain development. Verbal and numerical scales are highly applicable for one-dimensional postoperative pain assessment in most settings, which could help standardise pain assessment measurements [35]. Although the pain intensity assessment scale is included in nursing documentation in most healthcare systems, further studies describe that nurses lack the knowledge, attitude, and skills necessary for effective pain management and do not consider the use of pain intensity measurement scales essential for assessing patient pain [30,36].

The agreement between nursing assessments and postoperative pain self-assessments of postoperative pain in this study may also be the result of good preoperative preparation of patients for pain. The patient typically undergoes a structured interview with an anaesthesiologist about previous anaesthesia, allergies, and previously used methods of postoperative analgesia. The patient comes into contact with a nurse on the ward on the day before or the day of the surgical procedure. Despite the overall physical preoperative preparation and unfavourable staffing organization of the ward, the nurse usually manages to talk to the patient about the expectations and fears of pain and inform them about pain management options. Every patient has the right to be informed and educated, and with hospital accreditation, informing patients becomes a legal obligation for all employees [30]. Moreover, Wøien [37] reported that establishing evidence-based protocols for pain evaluation and documentation led to improved pain control plans. Despite the lack of significant differences between nursing evaluations and patient self-assessments of postoperative pain in this study, deeper analyses indicate interesting differences related to the scale’s extreme limits—that is, the estimated level of mild pain (number 3 on the NRS) and the level of severe pain (numbers 7 and 8 on the NRS). A higher percentage of nurses compared patients who assessed pain with a rating of 3 (NRS), while a higher percentage of patients rated their pain as 7 and 8 (NRS). Furthermore, the VRS scale also showed the greatest differences precisely in the marginal descriptions of pain, especially for the descriptions of “no pain” and “severe pain”, when a higher percentage of nurses assessed the patient’s pain indicating “no pain” and a higher percentage of patients indicated “severe pain”. Several studies describe differences between patients’ and nurses’ perceptions of patients’ pain based on pain assessment scales [38,39]. Research also describes significant differences in the perception of “moderate and extremely severe” postoperative pain, with nurses often underestimating postoperative pain compared to patients [38,39].

In this study, the pain was assessed as “unpleasant” on the VRS scale by 35.9% of nurses and 35% of patients. The highest agreement on the assessment of pain on the NRS scale in this study was in the selection of level 6. Choosing the “middle ground” in assessing the intensity of a patient’s pain by nurses creates a certain level of certainty in avoiding errors in assessment. When nurses deviate from the average value, they often assess and mark a lower level of pain. In descriptive scales, the occurrence of the “middle ground” is more common because data are obtained during the conversation, often without directly formulated questions about the pain. Non-verbal communication as well as many factors that affect verbal communication during the first postoperative day (e.g., fear, uncertainty, shame, and drowsiness) can favour the assessment. The literature describes an underestimation of a patient’s pain level on surgical wards that is often a result of insufficient nursing communication with the patient about pain. Nurses do not generally enquire about a patient’s subjective experience of pain. The experience of pain should be fully examined, as well as the meaning of pain to each patient. Experience in practise indicates that nurses on surgical wards are responsible for assessing and transmitting data about a patient’s pain to the doctor. In contemporary hospital practise in Croatia, the patient has a precisely determined dose of medicine written on the so-called therapeutic list by the operating surgeon or anaesthesiologist. Regardless of the intensity of pain expressed by the patient, the nurse gives the prescribed dose of medication to the patient as needed and monitors its effects and the patient’s reactions, along with a qualitative and quantitative assessment of pain. Objective pain measurement with a pain scale and adjustment of the therapeutic dose according to the result are rarely performed.

Continuous use of pain scales would improve patient understanding, safety in hospital treatment, nursing documentation, pain history tracking, standardisation of pain management procedures, and faster postoperative recovery, ultimately reducing healthcare costs. Although this study did not record significant differences in overall pain assessment between nurses and patients, differences were noted only at certain levels of pain. Constant monitoring and evaluation of pain intensity using pain scales would significantly improve the outcomes of pain management on surgical wards and contribute to positive changes in the approach of the entire healthcare team (patients with accurate labelling, nurses with assessment and documentation, and doctors with pain therapy management for pain). Fletcher et al. [40] focused their research on the frequency of severe postoperative pain in patients who evaluated their pain before and after surgery. They described that pain assessment in most patients in surgical wards was recorded in four-hour intervals, and in intensive care units, overall assessment was recorded in very few patients due to poor management of nursing documentation [40]. In fact, nurses’ non-cooperation is a possible problem in practise, because filling out yet another form of documentation at a time when there is an evident lack of nurses and increased workload causes reluctance. Allen et al. [10] concluded in their research that the likely causes of ineffective treatment of acute postoperative pain are insufficient knowledge of the pharmacodynamics and pharmacokinetics of drugs, generalised prescribing regimens, inadequate documentation, and disregard for individual needs and differences among patients. In addition to the insufficient readiness of the healthcare team to introduce pain measurement scales in all hospital wards, there is an additional obstacle for nurses in pain assessment and management. Today, in Croatian hospitals, nurses on patient wards work outside of their acquired competencies [41]. This study involved 81.5% of VET nurses, of whom 91.7% independently care for patients on surgical wards 24 h a day. It is possible that due to lower levels of knowledge, skills, autonomy, and responsibility, they are often uncertain about giving parenteral analgesic therapy since the responsibility remains with them and without legally regulated support [41]. Van Dijk et al. [42] described similar results in their study, in which they stated that nurses should not strictly adhere to the prescribed strong analgesic therapy in patients with tolerable pain but should assess the pain more frequently and treat it accordingly. Phuong Hoang Vu investigated organisational deficiencies and nursing barriers to proper assessment and effective pain management, citing the barriers nurses face as the main reasons for poor pain management, such as lack of professional staff, legal and institutional barriers (defining competencies and shared procedures), inadequate prescription of analgesics, and the inaccessibility of physicians or teams needed to review therapy [43]. It is important that nurses objectively measure patients’ pain using a measurable tool as much as possible to introduce new proposals to ensure good pain management in surgical wards [43]. In summary, differences in the assessment of the level of pain between nurses (who underestimate pain) and patients may be due to the subjectivity of pain assessment, different experiences with pain, different attitudes toward pain, and lack of communication. It is important for nurses to be trained in pain assessment as well as to establish good communication with patients to gain a comprehensive understanding of the patient’s pain experience and provide effective pain control.

### 4.2. Differences in Nursing Assessments and Patient Self-Assessments of Postoperative Pain Regarding the Characteristics of Respondents

In this study, the majority of VET-educated nurse respondents rated the pain as moderate on the VRS. Master’s-degree-holding nurses rated pain with a score of 6 on the NRS, while nurses with other levels of education rated pain with a score of 3. The differences in the perception of pain by nurses based on their educational background were greater on the NRS scale and smaller on the VRS scale, indicating high consistency among nurses in their work, as well as better marking on the NRS scale. On the verbal scale, MSc nurses did not rate patients as having any pain or unbearable pain in any case. As many as 77.7% of nurses in this study work in 12-h shifts (day and night) shifts, with VET education level and without BSc- or MSc-level supervisor education. These results suggest the expertise of nurses who continuously monitor and evaluate patients throughout the postoperative recovery period, as well as the high level of empathy of nurses regardless of their level of education. MSc nurses have more formal training, training, and clinical experience in pain management than VET nurses. This experience can help better understand and assess postoperative pain in patients. In some organisations, MSc nurses may be more involved in pain management and have more opportunities to specialise in that area, while in other organisations, this may not be the case [29]. MSc nurses may have more skills in communicating with patients and asking relevant questions about pain, enabling them to better assess and manage pain [29]. However, it is important to note that VET nurses are important in providing quality care to patients and they can also have the skills and knowledge needed to assess and manage pain. The results of this study indicate that there is no significant difference in the nursing assessment of postoperative pain on the NRS and VRS scales, depending on the length of work experience. The results obtained indicate that nurses, regardless of their work experience, assess pain well and are sufficiently professional and empathetic toward the patient. A similar study describes how patients accept nursing authority and expertise regardless of their years of work experience [30]. In this study, the largest number of nurses with 21–30 years of work experience rated the pain as “mild”, while nurses with less than 5 years of experience rated the pain as “moderate” on both scales.

In this study, male and female patients self-assess pain equally on the NRS scale, while the biggest differences in self-assessment of pain, based on sex, are on the VRS scale in the descriptions of “mild pain” and “severe pain”. However, the majority of patients on the VRS scale self-assess pain as moderate, unpleasant pain, especially men, which could be interpreted as patients objectively marking pain on the NRS scale because they can describe pain on the VRS scale. More men chose moderate pain on the VRS scale, but there are studies that describe men as more willing to endure and not express as much pain as they actually experience [32,44]. Wisea and colleagues [45] reported in their study that men were more willing to endure stronger pain than women but women talked more about the character of their pain than men; women are willing to express the pain in spontaneous conversation without additional targeted questions or numerical scale presentation [45]. Furthermore, Yang et al. [46] explained the importance of gender roles (identification with gender) in assessing expected behaviour and pain experience, as well as real behaviour when pain is present. The results clearly showed that there is an interaction between gender and gender identification in assessing how someone should or will tolerate pain. With low identification, there is no difference in expected and actual tolerance, but with high identification with their gender, the differences are significant [46]. Since the patients in this study have acute pain, it is important to simplify the chosen scale and standardise it for all patients.

In this study, it was found that the higher the assessment and self-assessment of postoperative pain on the NRS, the higher the VRS values in both groups of participants. Ho-Jin Lee and colleagues [35] reported similar results in a study on the one-dimensionality of postoperative pain intensity assessment between verbal, visual, and numerical scales. Compared to the visual and verbal scales, the numerical scale had the highest consistency and is mentioned as the most recommended tool due to its ease of use and good applicability, unlike the verbal and visual scales [35]. There are numerous studies that have investigated the correlation between numerical and verbal scales for pain assessment [17,18,35,47]. Although these two scales are different in nature, there is a strong correlation between them [35,47]. When a patient self-assesses pain on a numerical scale, they are actually assessing pain intensity. However, when pain is assessed on a verbal scale, the patient is trying to describe their painful sensation [47]. Therefore, if the intensity of pain is higher, it will most likely be reflected in the patient’s description of pain. For example, if a patient has intense pain, they will probably use words such as “very painful”, “unbearable”, etc. In short, as pain assessment increases on the numerical scale, pain assessment on the verbal scale is also likely to increase. These two scales complement each other in assessing the patient’s pain level and are used together to obtain a more accurate picture of the patient’s pain level. Ensuring effective pain therapy raises the reputation of hospitals in the public eye [1]. Quality assurance programs must include an assessment of the quality of healthcare (effectiveness and monitoring of indicators, adverse events), recognition of problems, and analysis of weak points by comparing them to standards. After analysis, proposals are necessary for overcoming the problems and continuous monitoring of success [19]. There are several components of quality assurance for pain management [1], such as general healthcare conditions that include the qualification of healthcare professionals, the quality of the equipment, and organisational financial opportunities of the organisation. Quality assurance requires registration of side effects and complications, monitoring and documentation of pain intensity, latency time from the onset of pain to the start of treatment, and the success of pain treatment [19].

### 4.3. Limitations and Recommendations for Future Research

In this cross-sectional study, participants are nurses who work in direct patient care and are employed in a clinical hospital in Croatia, as well as patients hospitalised in surgical departments during the postoperative period. Therefore, the sample is not large enough to generalise the results to all healthcare professionals and surgical patients in hospital institutions at the level of the Republic of Croatia. In the future, a study is planned to be conducted with more participants from different categories of hospital institutions (general and clinical centres) from different regions in Croatia. In addition, future studies could include the results of pain assessment after different types of surgery, as pain and its assessment method may differ depending on the type of surgery. Furthermore, different pain assessment techniques could be included and the effectiveness of different interventions in reducing postoperative pain could be investigated. Ultimately, possible cultural differences in pain assessment and self-assessment could be included and taken into account in the analysis of the results. This would contribute to a higher level of objectivity and a better understanding of the patient’s postoperative pain in the hospital environment.

### 4.4. Usefulness and Applicability of Study Results

The results of this study may have implications for the development of a higher quality treatment of patients’ pain after surgical procedures in surgical departments. Namely, the conducted study showed that nurses assess pain similarly to patients on two different one-dimensional scales. With their knowledge, empathy, and communication with patients, as well as the recognition of pain episodes in patients, nurses are ready to provide patients with an individualised approach to pain assessment and management after surgery. Based on the results of this study, hospital protocols can be established in the field of pain management related to the regular and continuous use of pain assessment scales and the individualisation of analgesic prescriptions for each patient. In addition to the abovementioned changes that the hospital could devise and implement, it is necessary to involve hospital management and physicians who are responsible for prescribing analgesia doses. The introduction of individualised pain management would contribute to meeting the needs of patients in terms of pain relief, which would ultimately significantly contribute to more effective overall patient care.

## 5. Conclusions

The results of this study indicate that there is no significant difference between nursing assessments and patients’ self-assessments of postoperative pain. The length of nursing work experience is not related to the level of assessed pain. Differences in pain assessment between nurses and patients were found in the group of nurses with 21–30 years of work experience, where there is a stagnation in professional enthusiasm and a decrease in willingness to work and collaborate with the team. The level of training of nurses is not related to the assessment of pain in patients. VET nurses are the ones who care for patients in this study for the highest percentage of 24 h, and their work with the patient does not diminish the quality of the patient’s pain assessment and, thus, overall health care. Nurses, regardless of work experience and professional qualifications, are sufficiently educated and empathetic to work with patients during the postoperative period. Consistent use of pain assessment scales, structured patient education with written materials, and individualisation of prescribed analgesia for patients will contribute to patient safety and satisfaction, as well as improve the overall quality of healthcare.

## Figures and Tables

**Table 1:** Sociodemographic characteristics of the respondents (*n* = 206).

Characteristics of Nurses		Number (%)
Gender	Male	8 (8)
	Female	95 (92)
Level of education	VET	84 (81.6)
	BSc	16 (15.5)
	MSc	3 (2.9)
Work experience (years)	<5	17 (16.5)
	6–10	14 (13.6)
	11–20	26 (25.2)
	21–30	28 (27.2)
	>30	18 (17.5)
Working hours	8 h morning shift	23 (22.3)
	12 h day/night	80 (77.7)
Characteristics of Patients		
Gender	Male	91 (44)
	Female	115 (56)

**Table 2:** Differences in nurse assessments and patient self-assessments of postoperative pain on the NRS scale with respect to the characteristics of the respondents.

Characteristics of Nurses		NRS Scale Values Median (IQR)	*p*
Gender	Male	2 (1.5–5.5)	0.63 *
	Female	3 (2–5)	
Level of education	VET	3 (2–4)	0.07 ^†^
	BSc	3 (2–5)	
	MSc	6 (5–6)	
Work experience (years)	<5 years	2 (1–4)	0.42 ^†^
	6–10	2.5 (2–3)	
	11–20	3 (2–5)	
	21–30	3.5 (2–5)	
	>30 years	4 (3–5)	
Characteristics of Patients			
Gender	Male	4 (2–5)	0.68 *
	Female	4 (2–5)	

IQR—Interquartile range; * Mann–Whitney U test; ^†^ Kruskal–Wallis test.

**Table 3:** Differences between nurse assessments and patient self-assessments of current postoperative pain intensity for individual descriptions of current pain intensity on the VRS scale.

Description of Current Pain Intensity on VRS	Nurses (*n* = 103)	Patients (*n* = 103)	Total (*n* = 206)	*p* *
	Number (%) of Respondents			
None	25 (24)	18 (17)	43 (21)	0.30
Mild	29 (28)	27 (26)	56 (27)	0.76
Moderate	37 (36)	36 (35)	73 (35)	0.99
Strong	11 (11)	21 (20)	32 (16)	0.08
Worst pain	1 (1)	1 (1)	2 (1)	0.99

* Fisher’s exact test.

**Table 4:** Differences in nurses’ assessments and patient self-assessments of the current intensity of postoperative pain on the VRS scale with respect to the characteristics of the respondents.

	VRS Scale Values						*p* *
	None	Mild	Moderate	Strong	Worst Pain	Total	
	Number (%) of Respondents						
**Characteristics of Nurses**							
Gender							0.89
Male	2 (8)	3 (10)	2 (5)	1 (9)	0	8 (7.8)	
Female	23 (92)	26 (90)	35 (95)	10 (91)	1 (100)	95 (92)	
Level of education							
VET	20 (80)	26 (90)	31 (84)	6 (55)	1 (100)	84 (81.6)	0.18
BSc	5 (20)	3 (10)	4 (11)	4 (36)	0	16 (16)	
MSc	0	0	2 (5)	1 (9)	0	3 (3)	
Work experience (years)							
<5 years	6 (24)	4 (14)	7 (19)	0	0	17 (16.5)	0.83
6–10	4 (16)	5 (17)	4 (11)	1 (9)	0	14 (14)	
11–20	6 (24)	6 (21)	9 (24)	5 (45)	0	26 (25)	
21–30	6 (24)	10 (34)	8 (22)	3 (27)	1 (100)	28 (27)	
>30 years	3 (12)	4 (14)	9 (24)	2 (18)	0	18 (17.5)	
**Characteristics of Patients**							
Gender							
Male	8 (17.8)	11 (24.4)	18 (40)	8 (17.8)	0	45 (43.7)	0.68
Female	10 (17.2)	16 (27.6)	18 (31)	13 (22.4)	1 (1.7)	58 (56.3)	

* Fisher’s exact test.

## Data Availability

All data generated analysed during the current study are available from the corresponding author on reasonable request.
